# Sulfonamide antibiotic reduction in aquatic environment by application of fenton oxidation process

**DOI:** 10.1186/1735-2746-10-29

**Published:** 2013-04-09

**Authors:** Somayyeh Dehghani, Ahmad Jonidi Jafari, Mahdi Farzadkia, Mitra Gholami

**Affiliations:** 1 Department of Environmental Health Engineering and Center for Water Quality Research, Tehran University of Medical Sciences, Tehran, Iran; 2 Department of Environmental Health, Faculty of Medical Sciences, Tarbiat Modares University, Tehran, Iran

**Keywords:** Antibiotic, Oxidation process, Fenton reaction, Sulfamethoxazole

## Abstract

Presence of antibiotics in the environment may cause potential risk for aquatic environment and organisms. In this research, Fenton oxidation process was offered as an effective method for removal of antibiotic sulfamethoxazole from aqueous solutions. The experiments were performed on laboratory-scale study under complete mixing at 25±2°C. The effects of initial antibiotic concentration, molar ratio of H_2_O_2_/Fe^+2^, solution pH, concentration of H_2_O_2_, Fe^+2^ and reaction time was studied on the oxidation of sulfamethoxazole in three level. The results indicated that the optimal parameters for Fenton process were as follows: molar ratio of [H_2_O_2_]/[Fe^+2^] = 1.5, pH= 4.5, and contact time= 15 min. In this situation, the antibiotic removal and COD reduction were achieved 99.99% and 64.7-70.67%, respectively. Although, Fenton reaction could effectively degrade antibiotic sulfamethoxazole under optimum experimental conditions, however, the rate of mineralization was not completed. This process can be considered to eliminate other refractory antibiotics with similar structure or to increase their biodegradability.

## Introduction

In recent years, several pharmaceuticals have been detected in aquatic environment such as treated drinking water, surface water, groundwater, wastewater treatment plants (WWTPs) effluents and sludge [1,2]. Release of these chemicals in the environment can is of high concern for public health, and may have undesirable health effects on humans, animals and ecosystem [2]. Antibiotics are such materials that can reach the environment via different routes like: human or animal excretions, pharmaceutical manufacturing plants effluents, medical wastes, animal fertilizer, municipal WWTPs and hospital wastewater [2,3].

Antibiotic sulfamethoxazole (SMX) is one of the most frequent sulfonamides in municipal wastewater [4]. This compound is persistent against conventional and biological treatments and its removal efficiency in WWTPs is moderately low [5,6]. It has been reported in WWTPs effluents up to 1.9 μg/L and can also be detected in drinking water in low range of ng/L [6]. SMX can stay in the environment more than one year and may cause problems like bacterial resistance [6]. Occurrence of bacterial resistance was observed to many types of antibiotics like ciprofloxacin, sulfamethoxazole, trimethoprim and vancomycin in hospital and municipal wastewater in Hamedan city, Iran [7].

Advanced treatment methods such as membrane processes, ozonation and activated carbon, have been considered to be more efficient to remove some pharmaceuticals [3]. Membrane techniques are not advisable, because of investment costs, required pretreatment of WWTP effluent and generation of concentrated side streams [3]. Advanced oxidation processes (AOPs) can be investigated as an appropriate option for pharmaceutical wastewater treatment [2,8-10]. Ozonation has been applied in eliminate some pharmaceuticals, but by-products in ozonated effluent are poorly characterized [3]. The main concern of ozonation for antibiotic degradation is conversion potential of this material to intermediate organic compounds and more resistant products to degradation [4]. Among AOPs, Fenton oxidation process has been gained attention with respect to treating wastewater containing hazardous organic chemicals [8]. In this process, decomposition of organic compounds occurs in the short time due to produce hydroxyl radicals (OH^**.**^) [11]. Some characteristics of Fenton reaction include (a) high performance, (b) simple technology, (c) low cost, (d) application of reagents with low toxicity [10] and (e) be effective in degradation of toxic and non-biodegradable pollutants [11]. Oxidation results of amoxicillin antibiotic by Fenton indicated that this compound can completely remove under optimal conditions of temperature, hydrogen peroxide and ferrous ion after 30 min reaction time [10]. Also, it has reported that Fenton reaction and ozonation enable to eliminate over 90% oxytetracycline from manure [12].

However, some studies have been investigated application of AOPs for antibiotic removal, but only few researches considered sulfonamide antibiotics degradation using Fenton reagent. The authors couldn't find in the literature studies for SMX removal by Fenton application. So, the results in this field were scarce. Furthermore, in previous studies on other antibiotic removal by Fenton reaction, the optimal parameters in various antibiotic concentrations didn't separately suggested and data was not available. The novel additional data on the removal efficiency of Fenton reagent would contribute to an improved understanding of antibiotics degradation. The experimental results could make understanding the Fenton process as well as many practical aspects of its potential application.

The aim of this study was to determine the variation of SMX residual concentration in different experimental conditions of Fenton’s oxidation process (H_2_O_2_/Fe^+2^) and optimum values of affecting parameters on oxidation were described.

## Materials and methods

The antibiotic SMX (4-amino-*N*-(5-methylisoxazol-3-*yl*)-benzene sulfonamide, C_10_H_11_N_3_O_3_S) was obtained from Sigma–Aldrich. Ferrous sulfate (FeSO_4_ .7H_2_O), hydrogen peroxide solution (30% w/w), H_2_SO_4_, NaOH, acetic acid, K_2_Cr_2_O_7_, HgSO_4_, Ag_2_SO_4_ and potassium hydrogen phethalate were purchased from Merck. HPLC grade acetonitrile was applied from Caledon. The chemical oxygen demand (COD) was measured using COD reactor HACH DRB200 and CECIL Aquarius spectrophotometer [13]. To adjust pH, the pH meter HACH HQ40d model was used. The concentrations of antibiotic SMX was determined with a CECIL HPLC (High performance liquid chromatography) with a UV detector and column: C18 (250 mm×4.6 mm I.D.) and elution was carried out using gradient mode. Mobile phases were acetonitrile and 0.5% acetic acid aqueous solution (v/v). Antibiotic was detected using UV absorbance at 272 nm [14]. The experiments were performed on laboratory-scale study in 250 mL glass reactor under complete mixing and at 25±2°C. The reaction solution was prepared with different concentrations of antibiotic SMX (0.079, 0.19 and 0.47 mM (millimolar)) and was subjected to Fenton treatment. Degradation of antibiotic during Fenton oxidation was considered under experimental conditions include: pH (the values of 2.5, 3.5, 4.5 and 6.5), molar ratio of [H_2_O_2_]/[Fe^+2^] (in 1.5:1, 3.5:1 and 5.5:1), [H_2_O_2_] (in 1.47, 2.94 and 4.41 mM), [Fe^+2^] (in 0.98, 1.96 and 2.94 mM) and reaction time (15, 30 and 60 min). To initiate experiments, the pH of reaction solution was adjusted. Then required amounts of hydrogen peroxide and ferrous sulfate were delivered into reactor in the batch mode. The samples were withdrawn at selected reaction times and analyzed by HPLC [14]. The COD reduction and DO/pH changes were also considered under optimal conditions. Determination of optimal parameter in each step was based on "one factor at a time" and all experiments were run in duplicate (in 90 runs). The results obtained were analyzed applying SPSS software by analysis of variance (ANOVA).

## Results and discussion

### Effect of molar ratio of H_2_O_2_/Fe^+2^

**Figure 1:**
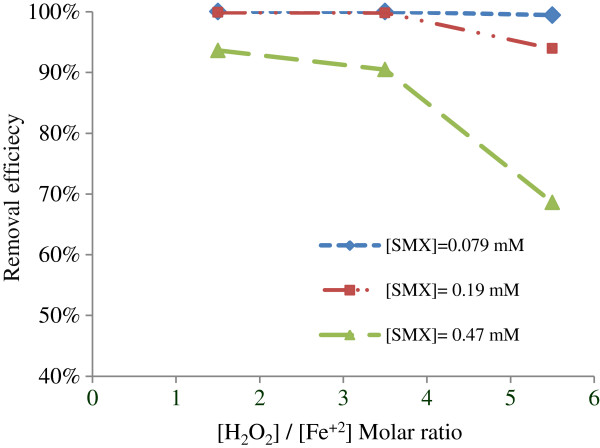
**Effect of [H**_**2**_**O**_**2**_**]/[Fe**^**+2**^**] molar ratio on antibiotic removal: [H**_**2**_**O**_**2**_**] = 1.47 mmol/L, [Fe**^**+2**^**] = varying, pH**_**o**_**= 2.5, retention time= 15 min.** The standard deviation of all data points was below 3.5.

The effect of [H_2_O_2_]/[Fe^+2^] molar ratio on Fenton efficiency in all concentrations of SMX (0.079, 0.19 and 0.47 mM) was illustrated in Figure 1. As shown, the highest antibiotic degradation (99.99%, 99.87% and 93.63% respectively) obtained in molar ratio of 1.5:1. In other ratios study, increasing this parameter from 1.5:1 to 5.5:1 reduced the SMX removal (P<0.039). It was found that [H_2_O_2_]/ [Fe^+2^] molar ratio had an important role in Fenton's removal efficiency. So that, with increasing the molar ratio (more than 1.5:1), SMX removal decreased due to reaction of additional H_2_O_2_ with OH^**.**^ radicals and production of weaker radicals with lower activity (Eq. 1) [15,16]. Also, self degradation of H_2_O_2_ to water and oxygen could be occurred (Eq. 2) [15].

(1) $$H_{2}O_{2}+{\mathrm{OH}}^{o}\to {\mathrm{HO}}^{o}{}_{2}+H_{2}O$$

(2) $$H_{2}O_{2}\to H_{2}O+O_{2}$$

Therefore, the molar ratio of [H_2_O_2_]/[Fe^+2^] was selected 1.5:1. Ben *et al*, was also obtained the same molar ratio in veterinary antibiotic removal by application of Fenton's reagents [17].

### Effect of Fenton reagents dosage

**Figure 2:**
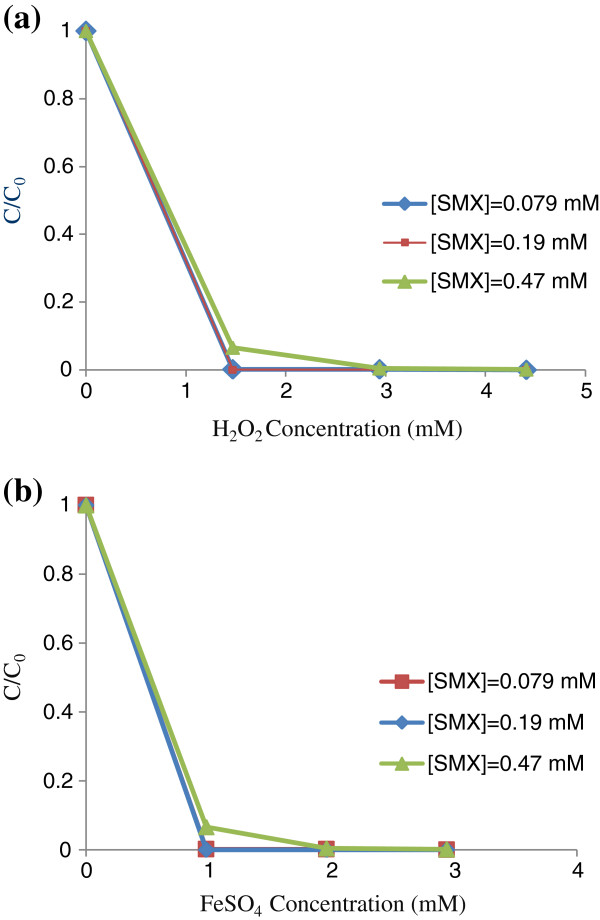
**Effect of different concentrations of (a) [H**_**2**_**O**_**2**_**] and (b) [FeSO**_**4**_**] on antibiotic removal in various concentrations: [H**_**2**_**O**_**2**_**]/ [Fe**^**+2**^**] = 1.5:1, [H**_**2**_**O**_**2**_**] = varying, [Fe**^**+2**^**] = varying, pH**_**o**_**= 2.5, retention time= 15 min.** The standard deviation of all data points was below 2.

Survey on the effect of different concentrations of Fenton reagents on SMX removal revealed that with increasing the [H_2_O_2_] and [FeSO_4_] (from 1.47 and 0.98 to 2.94 and 1.96 mM respectively), the process efficiency was enhanced in antibiotic concentrations study (Figure 2a,b). It means that higher simultaneous amounts of H_2_O_2_ and Fe^+2^ can improve oxidation potential of hydrogen peroxide by OH^**.**^[18]; Afterwards, hydroxyl radicals can break down organic compounds in a short time [18]. Additional amounts of reagents (more than optimum values) led to the less reduction variations (<0.5%). Because of more amounts of reagents can react with OH^**.**^ radicals and decrease the Fenton's efficiency [15,16,19].

### Effect of pH

In order to select the appropriate value of acidity, four levels of initial pH (2.5, 3.5, 4.5 and 6.5) were examined. Results showed that the antibiotic SMX was completely degraded (99.99% in all antibiotic concentrations) in pH of 3.5 to up (P>0.05). The Fenton process can operate well under acidic condition [17], but its function reduce in low pH because of slower FeOOH^+2^ formation and decrease production rate of Fe^+2^ and OH^**.**^[20]. Some researchers believe that remarkable reactivity may arise in higher pH value [19]. So that, investigation of clarithromycin removal by Fenton like process was considered at pH=7 as optimal value [21]. The results obtained in our study (99.99% removal efficiency in pH=6.5) emphasized this issue. It can be interpreted that antibiotic solubility and subsequently degradation efficiency was increased because of pH > pKa. Since the pH=4.5 was closest pH to reaction solution, it was considered as suitable amount.

### Effect of treatment time

Investigation of SMX reduction rate in different retention times (15, 30 and 60 min) demonstrated that 99.99% of SMX removal could be achieved at 15 min of reaction in all SMX concentrations study. So, retention time had direct influence on SMX removal by Fenton reaction. It should be noted that short retention time may involve higher chemicals consumption [18]. On the other hand, the long retention time can increase the reactor volume and constructional costs [18]. Considering the results, 15 min was chosen as appropriate oxidation time in this study. Survey have been done about degradation of amoxicillin based on Fenton process indicated that completely removal of antibiotic could be achieved after 30 min of reaction under optimum condition (pH=3.5, H_2_O_2_=3.5-4.28 mg/L, Fe^+2^=254-350 μg/L and temperature=20-30°C) [10].

### Effect of initial SMX concentration

The optimum conditions of Fenton reaction in each selected SMX concentration illustrated in Table 1. It was observed that initial SMX concentration had dramatic affect on process efficiency (P<0.009). So that, with the increment of antibiotic concentration from 0.19 to 0.47 mM was needed further amounts of H_2_O_2_ and ferrous ion. In the other words, required amounts of Fenton reagents depend on initial COD [15]. Meanwhile, in presence of higher amounts of organic compounds (antibiotic SMX), it's probably to increase competition of intermediate compounds with primary compounds [16]. This may be the reason of more required value of H_2_O_2_ and Fe^+2^ than lower SMX concentration.

**Table 1:** Optimal parameters of Fenton advanced oxidation process for SMX removal

**Antibiotic concentration (mM)**	**Molar ratio of [H**_**2**_**O**_**2**_**]/[Fe**^**+2**^**]**	**[H**_**2**_**O**_**2**_**] (mM)**	**[Fe**^**+2**^**] (mM)**	**pH**	**Reaction time (min)**
0.079	1.5	1.47	0.98	4.5	15
0.19	1.5	1.47	0.98	4.5	15
0.47	1.5	4.41	2.94	4.5	15

### COD and SMX removal

**Figure 3:**
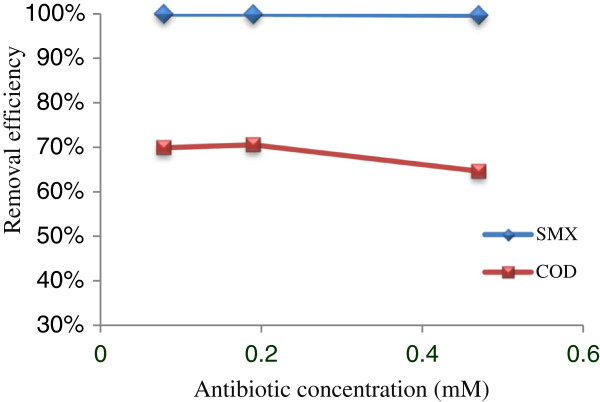
Removal efficiencies of antibiotic SMX and COD by Fenton process on optimal conditions in selected antibiotic concentrations.

The COD reduction rate compared with SMX removal in optimal conditions of Fenton process could be seen in Figure 3. As shown, COD reduction was smaller than antibiotic removal (64.7-70.67% and 99.99% respectively). It can be occurring due to inadequate hydrogen peroxide to oxidize entirely organic compounds [22]. Also, some of antibiotic SMX weren’t completely mineralized and may degrade to other organic compounds. In this regard, oxidation results of some pharmaceuticals using Fenton process showed that 56.4% of COD reduction could be obtained under operational conditions of pH=4, H_2_O_2_=3 M, Fe^+2^= 0.3 M and temperature=40°C during 10 min retention time [22].

### DO and pH variations

DO and pH variations were monitored during optimal conditions of Fenton process. It followed that DO and pH of reaction solution were gradually dropped from 6 to 2 mg/L and 4.5 to 3 respectively. The main variations of both parameters occurred within 5 min after initiating reaction. The reasons of rapid reduction in DO are high rate oxidation and degradation of majority organic compounds at beginning of reaction; Of course, oxygen produces during Fenton process and it increases again [20,23,24]. Decrease of pH can also be attributed to decomposition of organic compounds to organic acids [25]. DO and/or pH were studied in some researches and similar variation trend to oxidation progress were reported [20,23-25].

## Conclusion

In this study, degradation of antibiotic SMX by Fenton’s oxidation process was investigated and optimum values of affecting parameters on oxidation were described. Fenton’s reaction could effectively degrade antibiotic sulfamethoxazole (99.99% in all selected SMX concentrations) under optimum conditions. However the rate of mineralization was not completed (64.7-70.67% COD reduction). This process can be considered to eliminate other refractory antibiotics with similar structure or to increase their biodegradable. According to the purposes of treatment and effluent discharge standards, this process can be used as pretreatment of biological processes and/or post treatment of wastewater.

## Competing interests

The authors declare that they have no competing interests.

## Authors’ contributions

All authors participated to carry out this research and had surveillance on this article and its revisions. All authors read and approved the final manuscript.
